# Integrative Transcriptome and Proteome Analysis of the Tube Foot and Adhesive Secretions of the Sea Urchin *Paracentrotus lividus*

**DOI:** 10.3390/ijms21030946

**Published:** 2020-01-31

**Authors:** Robert Pjeta, Herbert Lindner, Leopold Kremser, Willi Salvenmoser, Daniel Sobral, Peter Ladurner, Romana Santos

**Affiliations:** 1 Institute of Zoology and Center of Molecular Biosciences Innsbruck, University of Innsbruck, 6020 Innsbruck, Austria; robert.pjeta@uibk.ac.at (R.P.); willi.salvenmoser@uibk.ac.at (W.S.); 2 Division of Clinical Biochemistry, Biocenter, Innsbruck Medical University, 6020 Innsbruck, Austria; herbert.lindner@i-med.ac.at (H.L.); leopold.kremser@i-med.ac.at (L.K.); 3 Departamento Ciências da Vida, Faculdade de Ciências e Tecnologia–Universidade Nova de Lisboa, Campus de Caparica, 2829-516 Caparica, Portugal; dv.sobral@fct.unl.pt; 4 Centro de Ciências do Mar e do Ambiente, Departamento de Biologia Animal, Faculdade de Ciências, Universidade de Lisboa, 1749-016 Lisboa, Portugal

**Keywords:** echinoderm, sea urchin, bioadhesion, differential RNAseq, mass spectrometry, in situ hybridization

## Abstract

Echinoderms, such as the rock-boring sea urchin *Paracentrotus lividus*, attach temporarily to surfaces during locomotion using their tube feet. They can attach firmly to any substrate and release from it within seconds through the secretion of unknown molecules. The composition of the adhesive, as well as the releasing secretion, remains largely unknown. This study re-analyzed a differential proteome dataset from Lebesgue et al. by mapping mass spectrometry-derived peptides to a *P. lividus* *de novo* transcriptome generated in this study. This resulted in a drastic increase in mapped proteins in comparison to the previous publication. The data were subsequently combined with a differential RNAseq approach to identify potential adhesion candidate genes. A gene expression analysis of 59 transcripts using whole mount in situ hybridization led to the identification of 16 transcripts potentially involved in bioadhesion. In the future these data could be useful for the production of synthetic reversible adhesives for industrial and medical purposes.

## 1. Introduction

Marine biological adhesives offer impressive performance in water, which is considered as the *enemy* of synthetic glues. Biological adhesives have a huge potential to inspire the development of a new generation of biological superior adhesives for an increasing variety of high-technology applications.

Bioadhesion is vital for many aquatic animals. Through the production of adhesive secretions, they attach, move, feed and defend themselves in their habitats. Studying this phenomenon allows us to better understand this complex physiological process and to gather important information needed for the development of new wet-effective, biocompatible and ecological biomimetic adhesives for medical (e.g., surgical adhesives) and (bio-)technological (e.g., promoters of cellular adhesion for tissue engineering) applications.

Amongst aquatic bioadhesives, cements that permanently attach animals to the substrate are best studied. This is the case for mussels, barnacles and sandcastle worms [1,2,3,4,5,6,7]. Comparatively, animals with non-permanent adhesion, such as barnacle larvae, flatworms, cnidarians and echinoderms, have been much less studied, thus, their reversible adhesion is just beginning to be understood.

Barnacle cyprid larvae have strong (0.1–0.3 MPa) [8,9,10] but reversible adhesion [11,12]. Their bioadhesive is produced in different gland cells, being extruded through long, vesicle-filled necks up to the surface [9,12,13]. Thus far, there have been no reports on the existence of a releasing gland. The cyprid reversible adhesive is mainly composed of basic [14] and acidic [15] proteins. Thus far only one cyprid footprint protein has been characterized, settlement-inducing protein complex (SIPC), which presents three glycosylated subunits with apparent molecular weights of 98, 88 and 76 kDa [16] and an acidic pI of 4.6–4.7 [15]. Cloning of the cDNA encoding for SIPC in *Amphibalanus amphitrite* showed that this protein has 171.7 kDa [16], is glycosylated [17] and shares 30% of sequence identity with α-macroglobulin [18]. Recently, an orthologous protein named MULTIFUNCin was identified in *Balanus glandula*, sharing 78% nucleotide sequence identity with SIPC. It has 199 kDa and is also expressed as three subunits [19]. Both proteins are believed to perform multiple roles, namely, induce the permanent settlement of cyprids [20], function as a temporary adhesive [15] and direct the formation of the barnacle calcite basal plate during cyprid metamorphosis [21]. In recent years, several transcriptomic and proteomic analyses have revealed more candidate proteins involved in cyprid attachment [22,23,24,25,26] but their specific role remains unknown.

Free-living flatworms use a duo-gland adhesive system to rapidly adhere to and release from the substrate [27]. Their adhesive system consists of dozens to hundreds of adhesive organs, segregated in the tail plate or spread all over their body [27]. Each adhesive organ comprises two different gland cell types: adhesive gland cells, which are supposed to secrete the adhesive proteins and releasing gland cells, which produce de-adhesive molecules. Both glands protrude out of the epidermis surrounded by modified microvilli of an epithelial cell, which is called an anchor cell [28]. Recently, the adhesive systems of two free-living flatworms have been characterized [29,30]. The adhesion of *Macrostomum lignano* relies on two adhesive proteins, *M. lignano* adhesive protein 1 (Mlig-ap1) and *M. lignano* adhesive protein 2 (Mlig-ap2) [29]. Mlig-ap2, the adhesive protein, displaces water molecules from the substrate and promotes adhesion, whereas Mlig-ap1 has a cohesive function, connecting Mlig-ap2 to the microvilli of the surrounding anchor cells. Detachment is caused by the release of a small, negatively charged molecule that interferes with the positively charged Mlig-ap1, perturbing the adhesive cohesiveness [29].

In the proseriate flatworm *Minona ileanae*, the adhesive organs are cushion shaped and several branching adhesive and de-adhesive gland necks protrude out of one anchor cell [30]. In *M. ileanae*, five adhesive proteins (Mile-ap1 to 5) were identified. Their detailed roles are not known, but together, they accomplish the adhesive and cohesive function, as demonstrated in knock-down experiments [30]. 

Parasitic flatworms can attach to host surfaces through the secretion of a thin layer of adhesive material. Knowledge on attachment to living surfaces is scarce and is mostly based on histochemical studies [31].

Cnidarians, such as the freshwater polyp *Hydra magnipapillata*, are predominantly sessile but can detach voluntarily in response to environmental changes. However, in the attachment area, which is called the basal disc, only one gland cell type that produces the adhesive has been found [32]. Thus far, glycans and/or glycoproteins have been located in adhesive secretory granules using specific staining [32], as well as eight transcripts with glycan-binding functions, raising the possibility that the non-covalent cross-links contributed by glycan protein binding might be involved in the adhesive cohesion [33]. A transcript that comprised a chitin-binding domain was also specifically expressed in basal disc cells, indicating an ability to bind chitin (chain polymer of *N*-acetylglucosamine), which could be useful to crosslink with other structural adhesive proteins [33]. Peroxidase-like enzymes were also highly concentrated in basal disc cells, suggesting that they function as catalyzers of crosslinking within the adhesive or have an antioxidant or antimicrobial role [32,33]. The release is believed to be a combination of muscular contraction and enzymatic detachment supported by the identification of two types of protease inhibitors and one type of glycosylase (glycosyl hydrolase AbfB) exclusively expressed in basal disc cells [32,33].

**Figure 1:**
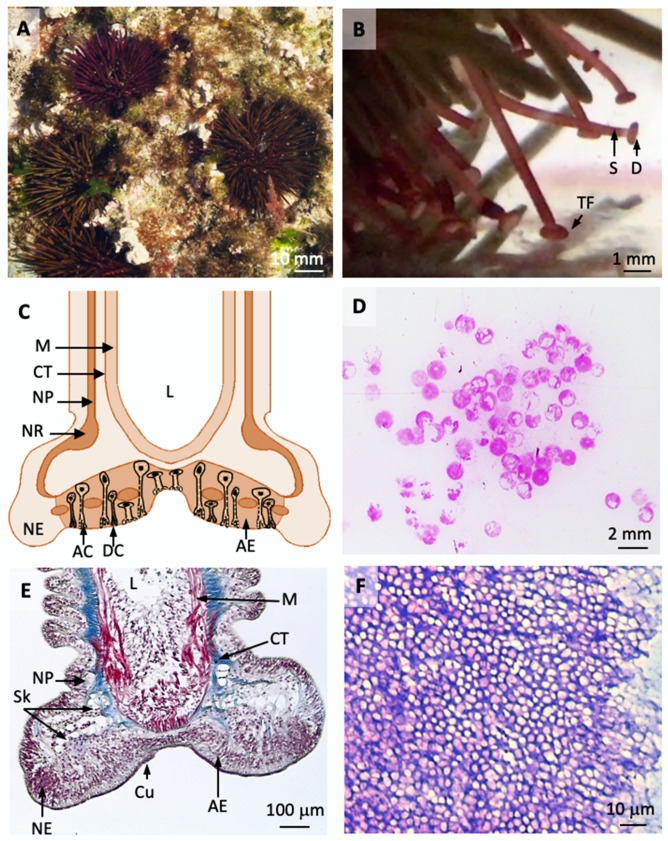
Rock-boring sea urchin *Paracentrotus lividus* (**A**) has hundreds of oral tube feet specialized for locomotion and adhesion (**B**). Tube feet have a proximal cylindrical motile stem and a distal flattened disc with a duo-glandular adhesive epidermis with adhesive and de-adhesive secretory cells (**C**,**E**). After detachment, circles of adhesive secretion remain attached to the substrate and can be visualized after staining with an aqueous solution of Crystal Violet (**D**,**F**). Abbreviations: AC, adhesive secretory cell; AE, adhesive epidermis; CT, connective tissue; Cu, cuticle; D, disc; DC, de-adhesive secretory cell; L, lumen; M, myomesothelium; NE, non-adhesive epidermis; NP, nerve plexus; NR, nerve ring; S, stem; Sk, skeleton; TF, tube feet.

Echinoderms, like sea stars and sea urchins have hundreds of specialized adhesive organs, called tube feet (Figure 1A,B), that attach strongly (0.1–0.5 MPa) but reversibly to the substrate [34]. These adhesive organs are flattened at their tip, forming an adhesive disc that encloses adhesive and releasing cells (Figure 1C,E) [35,36]. Adhesive cells can be of one or more types and have long necks ending as cuticular pores in sea stars or tufts of microvillar-like projections in sea urchins, through which the adhesive is delivered to the surface [35]. The three most commonly studied echinoderms in terms of their adhesive organs and footprint composition are the sea stars *Asterias rubens* and *Asterina gibbosa* [37,38,39,40,41,42] and the sea urchin *Paracentrotus lividus* [43,44,45,46]. 

In *A. rubens*, 34 footprint-specific proteins have been identified, but only 20 could be annotated [38]. Some candidate adhesive components such as lectin-like proteins are believed to promote adhesion to the biofilm present on the substrate because of their ability to bind glycans. Mucin-like proteins are possibly involved in the formation of structural networks through their potential ability to oligomerize and/or cross-link to other adhesive molecules. Peroxidases would allow the formation of cross-links between the adhesive proteins and thereby improve footprint cohesion. Proteins with hyalin, epidermal growth factor (EGF)-like and discoidin domains are known to mediate protein–protein, protein–carbohydrate or protein–metal interactions. Some releasing candidates have also been identified, such as proteases with metalloendopeptidase activity that could degrade adhesive proteins and consequently induce detachment [38]. The only fully characterized adhesive protein, named sea star footprint protein 1 (Sfp1), has a predicted molecular weight of 426 kDa and is auto-catalytically cleaved before its secretion into four subunits with 57, 231, 72 and 66 kDa. Sfp1 shares many characteristics with flatworm Mlig-ap1. Both are believed to have a cohesive role, share common carbohydrate- and metal-binding domains and have high cysteine content [29,39]. Recently, a comparative interspecific study revealed that Sfp1 is present in all sea star orders and in the representatives of all tube foot morphotypes, regardless of functional habitat adaptations [42]. However, most sequences found in the available transcriptomes were partial, and the Sfp1 full-length coding sequence was only found in three out of 18 studied species (*Pisaster ochraceus*, *Pteraster tesselatus* and *Patiria pectinifera*). It appears that sea star cohesive proteins are highly conserved amongst species, whereas the sequence of adhesive proteins varies considerably [42]. Glycans are also an important component of sea star adhesives. The adhesive footprints of *A. rubens* contain sialylated proteoglycans and two glycoproteins with galactose, *N*-acetylgalactosamine, fucose and sialic acid residues [40], whereas in *A. gibbosa* only α-linked mannose glycans have been detected [41].

In *P. lividus*, several proteome studies of the tube foot disc (Figure 1B,C,E) and the adhesive secretion (Figure 1D,F) indicated 328 non-redundant disc-specific proteins, of which 163 were highly over-expressed [43,44,45]. Only one of these proteins, Nectin, was shown to be present in the adhesive secretion, but whether it has an adhesive and/or cohesive role remains unclear [45]. This protein has 108.3 kDa, presents phosphorylated and glycosylated isoforms, and contains six discoidin-like domains (similar to Sfp1) that can bind molecules bearing galactose and *N*-acetylglucosamine residues [44,45,46]. Two heme-peroxidases highly expressed in the adhesive disc were also highlighted for their possible involvement in the polymerization of sea urchin adhesives. Other enzymes, such as hydrolases acting on peptide bonds and glycosyl groups, were also over-expressed in the adhesive disc and interpreted as putative release molecules. As for the glycan component of sea urchin adhesives, thus far, only indirect evidence has been reported, such as the presence of sulphated glycosaminoglycans, asparagine-oligosaccharides and sialic acids, provided by the identification of highly expressed sulfatases, asparaginases and sialidases in the adhesive disc [45].

Although there are still major gaps in our understanding of reversible adhesion, some common features that are a direct result of the increasing number of transcriptomic and proteomic data on non-permanent adhesives are beginning to emerge (Table 1). Some studies used a transcriptomic approach, providing information on all expressed transcripts even in the absence of protein information [25,38,42]. Complementing this approach with a differential transcriptome/proteome is advantageous to compare tissue containing and lacking adhesive cells [23,26,30,33,47], which, in turn, helps to identify differentially expressed transcripts/proteins on a qualitative and quantitative level, generating an adhesion-specific candidate gene/protein list [48]. 

In this study, we combined transcriptome sequencing of *P. lividus* tube feet, with differential gene expression analysis and in situ hybridisation (ISH). We also re-analyzed the previously obtained tube feet differential proteome and the secreted adhesive proteome [45] with a new *P. lividus* species-specific adhesion transcriptome. This approach allowed us to extend the list of transcripts/proteins specific of adhesive discs and adhesive secretions, identify novel adhesion-related proteins (i.e., with no annotation in public databases), perform a more confident annotation of proteins (through the use complete or partial open reading frames and not just a few peptides) and validate transcript expression in tube feet whole mounts and semi-sections.

## 2. Results

**Figure 2:**
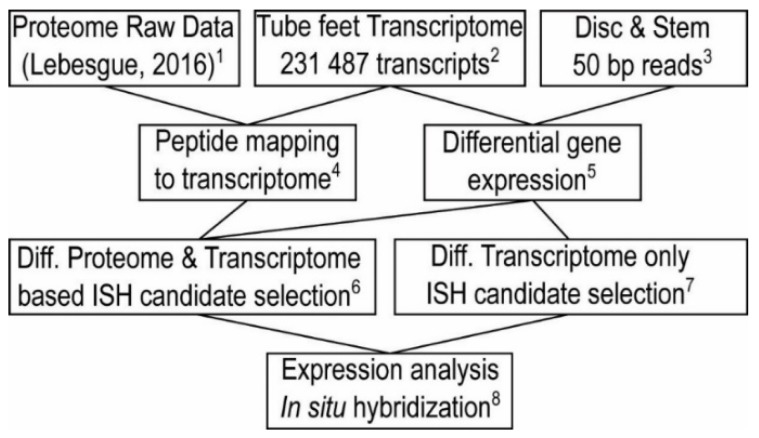
Summary diagram of the integrative transcriptomic and proteomic analysis of the present study. ^1^ Raw data of Lebesgue et al., containing 10 *P. lividus* disc-, eight stem- and three adhesive secretion samples, were used for the present study. ^2^ *P. lividus* tube feet transcriptome was generated. ^3^ Disc and Stem specific differential RNAseq reads were generated. ^4^ Re-mapping of the Lebesgue et al. proteome data to the new *P. lividus* transcriptome. ^5^ Identification of adhesive disc specific transcripts using DESeq2 differential gene expression analysis. ^6^ Selection of candidate transcripts for in situ hybridization (ISH), based on the differential proteome and differential transcriptome. Only transcripts present in both datasets were considered for the ISH screen. ^7^ In order to ensure an encompassing dataset of disc-specific transcripts, a selection of differentially expressed transcripts, not present in the proteome (due to e.g., insolubility or post-translational modifications), was included. ^8^ ISH screen of the 59 selected disc-specific transcripts.

A previous study [45] used a proteomic approach to identify the proteins involved in sea urchin reversible adhesion. This study used a quantitative approach to compare protein expression levels in the tube foot disc (adhesive part) versus the stem (non-adhesive part), in combination with the protein profile of the adhesive secretion. However, at that time, no sequencing data of *P. lividus* tube feet were available and mass spectrometry-derived peptides were mapped to publicly available sea urchin protein databases. This approach only allowed the identification of highly conserved proteins, whereas species-specific proteins could not be detected. In the present study, we combined transcriptomics, differential gene expression, re-mapping of proteomic data and an in situ hybridization screen to identify new adhesion-related candidates (Figure 2).

First, we generated a tube foot-specific transcriptome of *P. lividus*, which allowed a re-analysis of the previous proteome data [45]. This transcriptome consisted of 270,361 transcripts with a N50 of 1499 bp and a GC content of 37.26% (Table 2).

**Figure 3:**
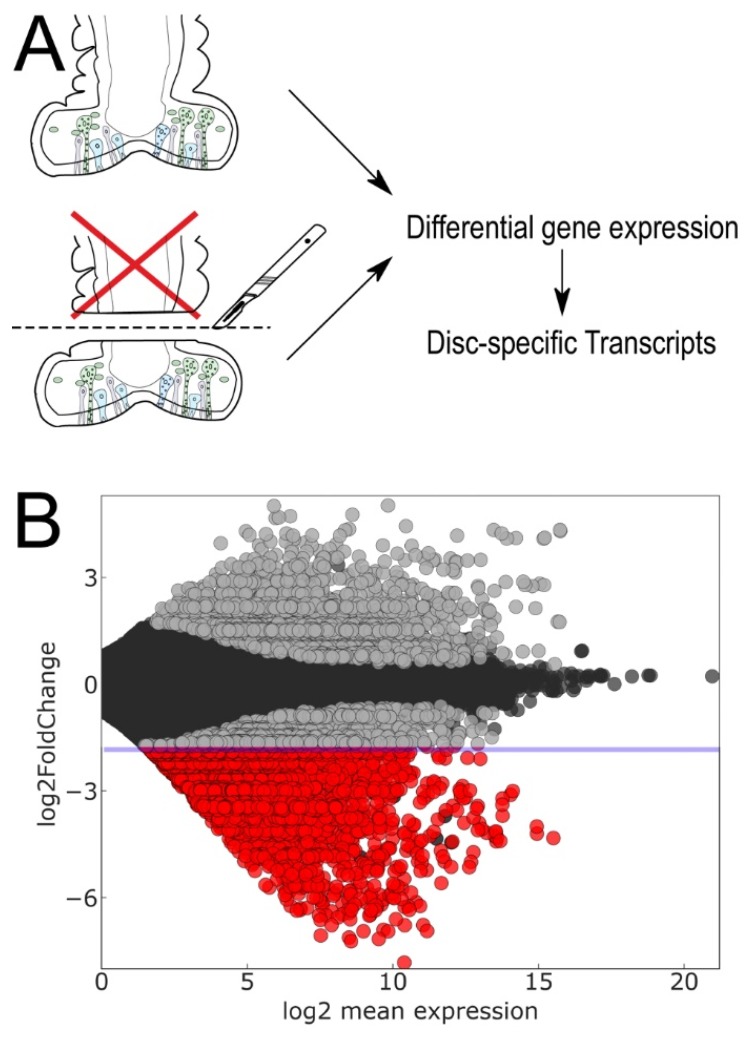
RNAseq and differential gene expression. Scheme (**A**) representing the differential gene expression approach. (**B**) Differentially expressed transcripts with an adjusted *p*-value < 0.01 are indicated in grey and red. 2129 disc-specific transcripts with a log2 fold change ≤ 2 are highlighted in red (see Table S1).

Second, we identified disc-specific transcripts using a differential RNAseq approach (Figure 3). Tube foot discs, which contained the adhesive and de-adhesive secretory cells, were separated from the stems. Disc and stem tissues were processed for RNAseq (Figure 3A) (see Materials and Methods). Differential gene expression analysis (Figure 3B) revealed 2129 transcripts over-expressed in the disc (≥four-fold) compared with the stem (log2 fold change ≤ −2; Figure 3B and Table S1).

**Figure 4:**
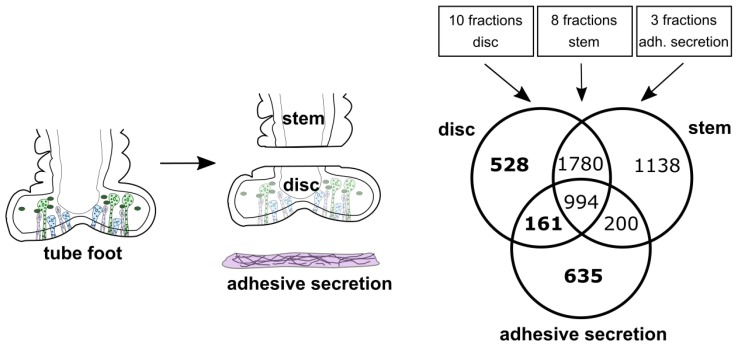
Illustration of biological samples used for differential proteomics and diagram indicating the number of transcripts allocated to disc-, stem- and adhesive secretion samples after re-mapping of the Lebesgue et al. proteome data to the *P. lividus* transcriptome. A total of 1324 transcripts (emphasized in bold) were used for downstream analysis.

Third, we performed a re-mapping of the previously obtained tube feet differential proteome and the secreted adhesive proteome [45] using the new *P. lividus* transcriptome. A total of eight stem fractions (Table S2, Tabs A–H), 10 disc fractions (Table S2, Tabs I–R) and three adhesive secretion fractions (Table S2, Tabs S–U) were re-mapped. Overall, 1324 disc- or adhesive secretion -specific proteins were identified. From these, 528 genes were exclusively identified in disc datasets (disc-specific), 635 in only in adhesive secretion datasets (adhesive secretion-specific) and 161 could be found in both, disc and adhesive secretion datasets (disc- and adhesive secretion-specific). (Figure 4 and Table S3).

**Figure 5:**
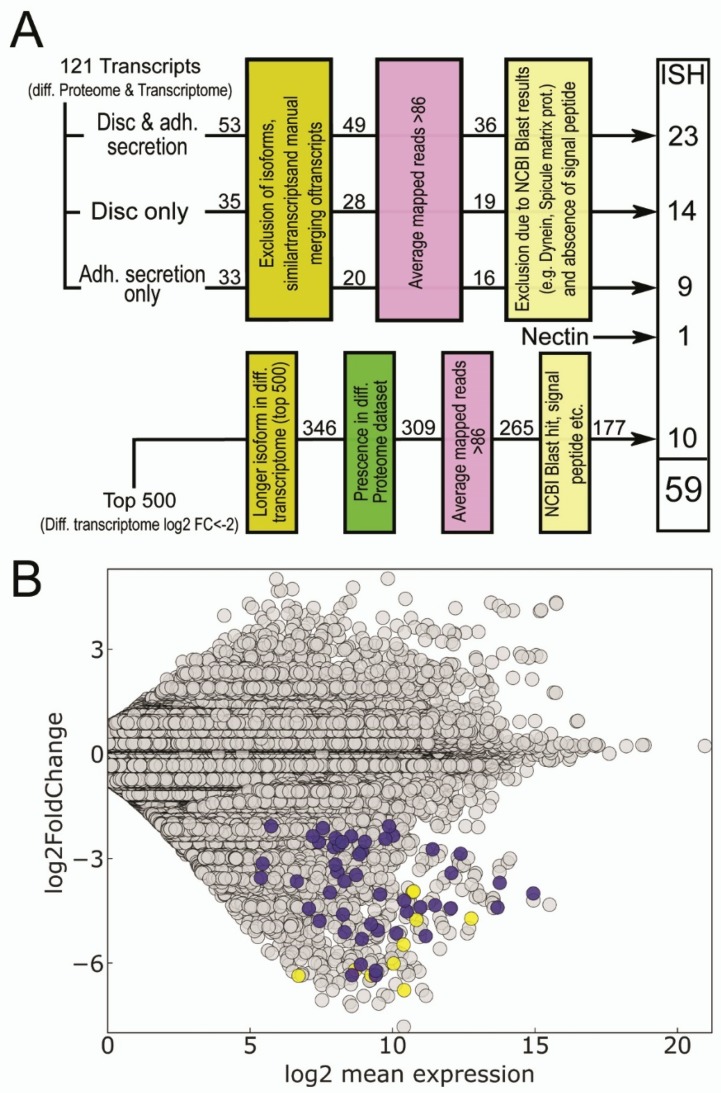
In situ hybridization candidate gene selection. (**A**) Candidate gene selection workflow. Initially, 121 transcripts were found in proteome data from disc and adhesive secretion samples as well as being over-expressed in the tube feet discs in the differential transcriptome. Exclusion of isoforms and similar transcripts, low expressed transcripts and exclusion of genes with non-adhesion related NCBI BLAST hit reduced in situ candidates to 49 transcripts. Additionally, 10 transcripts were selected for in situ hybridization that were differentially expressed but was not identified in the differential proteome dataset. (**B**) Differential gene expression analysis. Transcripts that were selected for in situ hybridization are highlighted in blue (differential proteome and differential transcriptome selection) and yellow (differential transcriptome only candidate selection).

Next, we searched for overlapping transcripts between the RNAseq analysis (2129 disc-specific candidates; Table S4, Tab A) and the proteome re-mapping (1324 disc- and adhesive secretion-specific hits; Table S4, Tab B). Overall, 121 transcripts were identified (Table S4, Tab C). From this subset (Figure 5A), we excluded low-expressed transcripts (Table S4, Tab D) and transcripts with a high sequence similarity (Table S4, Tab E). Furthermore, we removed transcripts with a clear non-adhesion-related BLAST result, such as spicule matrix protein or dynein (Table S4, Tab F). In addition, full-length candidates lacking a signal peptide were also excluded (Table S4, Tab G). One more transcript, Nectin, was added to the ISH screen. Although in the present differential gene expression analysis, Nectin log2 differential expression in the disc was only −1.68 relative to the stem (the selection criterion was log2 fold change ≤ −2; Table S3, Tab H), this protein was shown previously to be highly expressed in the differential proteome (up to 13-fold in the disc relative to the stem) and to be a part of the adhesive secretion [45,46]. To observe whether there are adhesive genes that are not present in the proteome data, but are highly expressed in discs in the differential transcriptome, we handpicked 10 transcripts from the 500 transcripts with differential expression in the discs (Table S5). Overall, a set of 59 transcripts were selected for an in situ hybridization screen (Figure 5).

**Figure 6:**
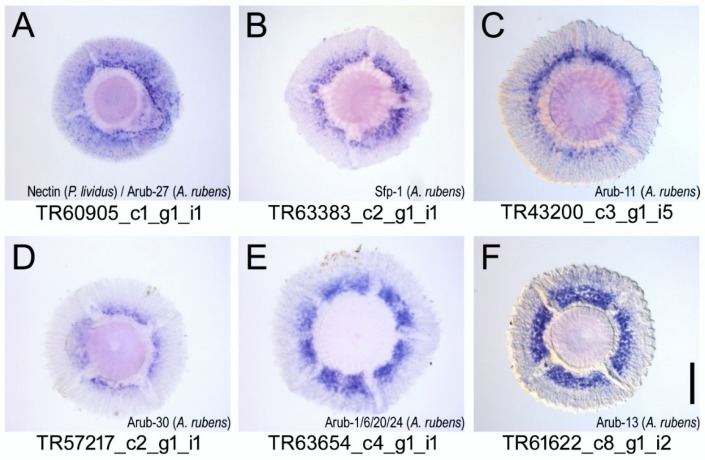
In situ hybridisation expression patterns of selected adhesion candidate genes previously identified in *P. lividus* (**A**) and in the sea star *Asterias rubens* (**B–F**). The respective BLAST hit for each transcript is shown on the bottom right of each image. Scale bar, 200 µm.

**Figure A1:**
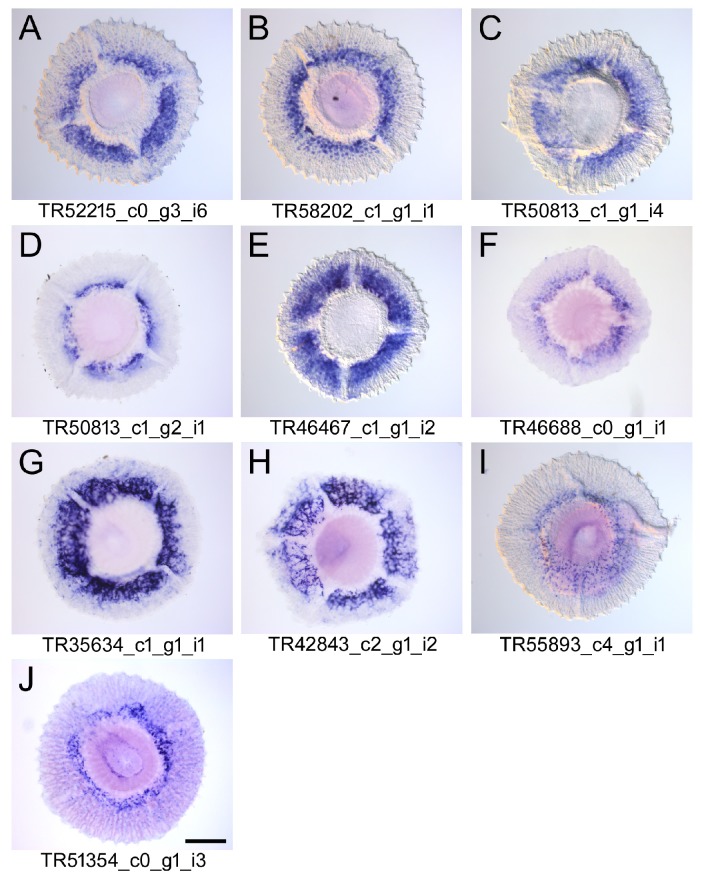
In situ hybridization screen panel 1. (**A**–**J**) Ventral view onto the tube foot disc. The distribution of the *in situ* hybridization signal (blue) resembles the localization of the adhesive gland cells bodies. Therefore, these transcripts potentially constitute proteins involved in bioadhesion. Scale bar, 200 µm.

**Figure A2:**
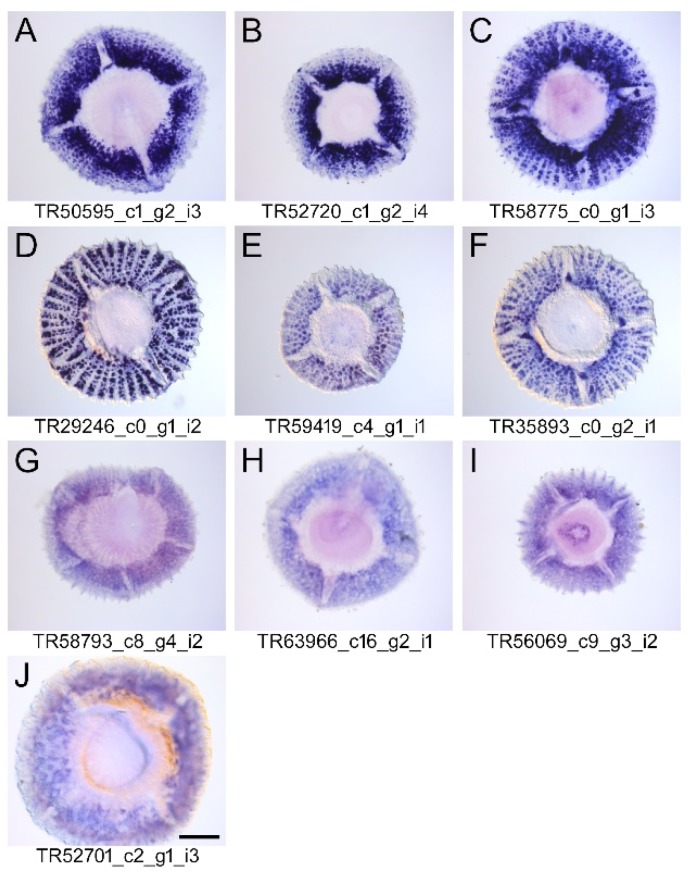
In situ hybridization screen panel 2. (**A**–**J**) Ventral view onto the tube foot disc. The distribution of the *in situ* hybridization signal (blue) resembles the localization of the adhesive gland cells bodies plus additional cells of the adhesive disc. Therefore, these transcripts potentially constitute proteins involved in bioadhesion or other proteins of the disc cells. Scale bar, 200 µm.

**Figure A3:**
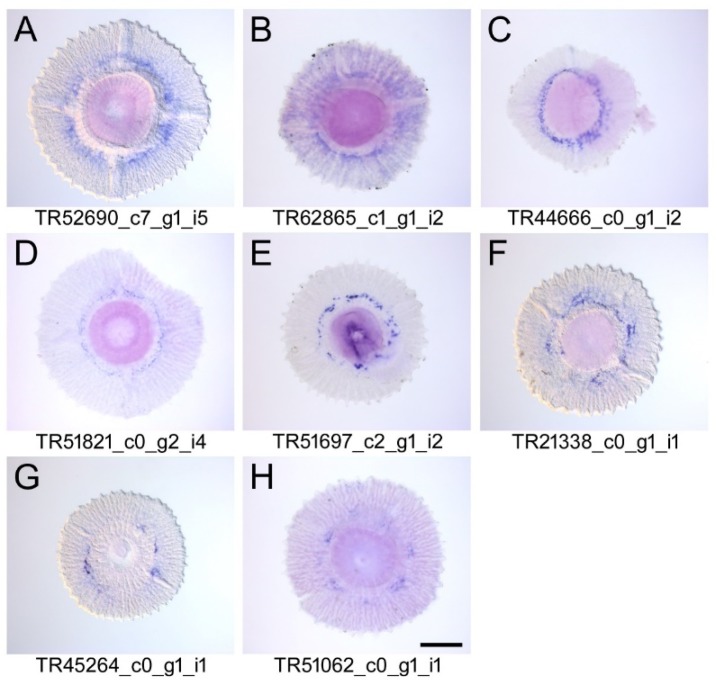
In situ hybridization screen panel 3. (**A**–**H**) Ventral view onto the tube foot disc. The distribution of the *in situ* hybridization signal (blue) is weak, but shows distinct expression in a subset of disc cells. Therefore, these transcripts represent proteins of unknown function. Scale bar, 200 µm.

**Figure A4:**
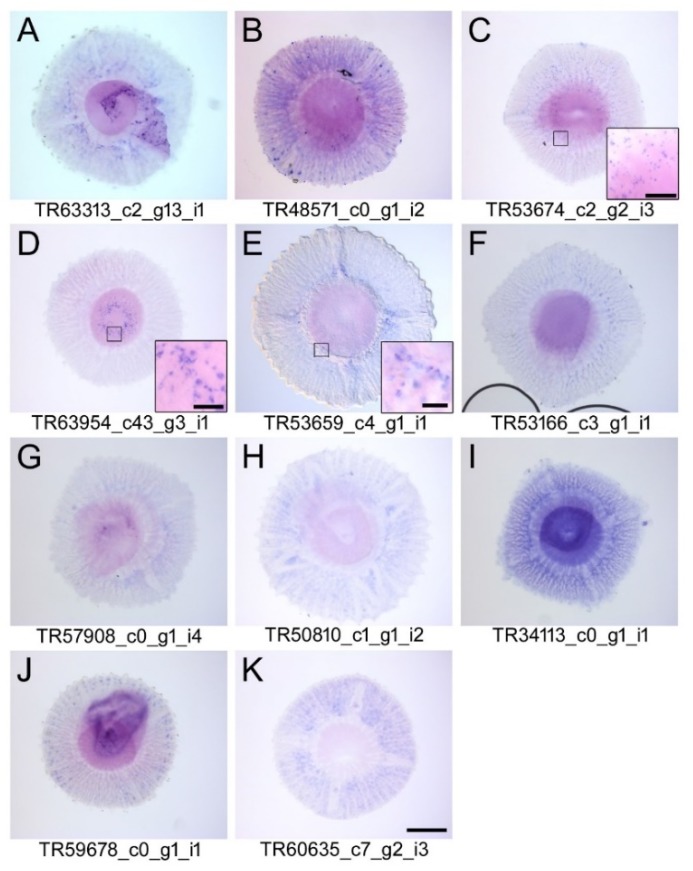
In situ hybridization screen panel 4. (**A**–**K**) Ventral view onto the tube foot disc. The distribution of the *in situ* hybridization signal (blue) is very weak and present only in a small subset of disc cells. Therefore, these transcripts represent proteins of unknown function. Scale bar, 200 µm, inset C, D and E, 25 µm.

Of the 59 transcripts selected for the in situ hybridisation, 45 showed an expression pattern in the adhesive disc (Figure 6 and Figure A1, Figure A2, Figure A3 and Figure A4). To identify which of these 45 transcripts were relevant adhesive candidates, we searched for adhesive gene orthologues that were recently identified in the sea star *A. rubens* [42] (Table S6). Out of 34 *A. rubens* adhesion-related transcripts, 16 had a BLAST hit to eight *P. lividus* transcripts selected for the in situ hybridization screen. Out of the eight *P. lividus* transcripts with homologous sea star adhesive candidates, six (TR60905_c1_g1_i1, TR63383_c2_g1_i1, TR43200_c3_g1_i5, TR57217_c2_g1_i1, TR63654_c4_g1_i1, TR61622_c8_g1_i2) exhibited an identical ISH expression pattern consisting of a pronounced ring-shaped labelling of the tube foot disc around the base of the stem, corresponding to the location of the adhesive secretory cell bodies (Figure 6). The remaining two *P. lividus* transcripts (TR48571_c0_g1_i2 and TR59872_c1_g1_i1) did not show a specific expression by ISH.

A similar ring-shaped expression pattern of the tube foot disc could be found in 10 more transcripts (TR52215_c0_g3_i6, TR58202_C1_g1_i1, TR50813_c1_g1_i4, TR50813_c1_g2_i1, TR46467_c1_g1_i2, TR46688_c0_g1_i1, TR35634_c1_g1_i1, TR42843_c2_g1_i2, TR55893_c4_g1_i1 and TR51354_c0_g1_i3), but homology to sea star adhesion candidates could not be identified (Figure A1). Therefore, given their similar expression pattern, specifically labelling the location of the adhesive cells, together with their putative functions based on the obtained BLAST hits and domain prediction, these 16 transcripts were considered new *P. lividus* adhesive candidates (Table S7).

The remaining 29 transcripts showed staining in both adhesive and non-adhesive areas of the tube foot disc (Figure A2) or other tissues not involved in the adhesive process (Figure A3 and Figure A4).

**Figure A5:**
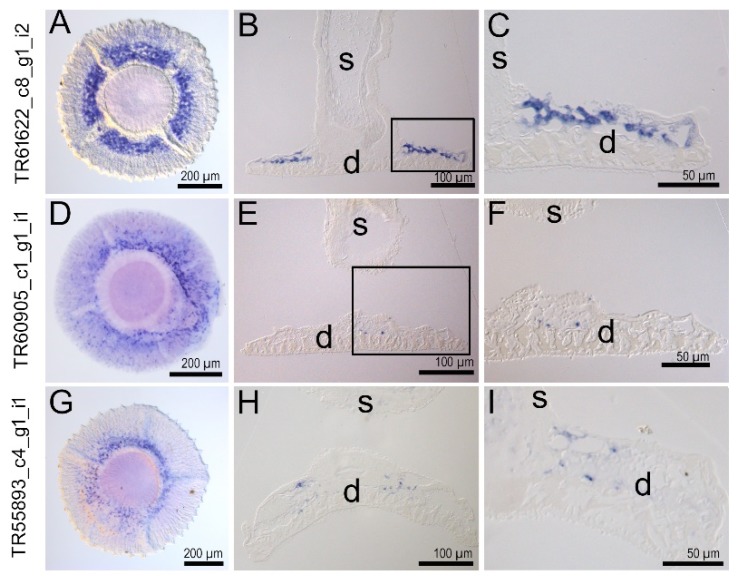
Spatial distribution of labeled cells within the tube foot disc. *In situ* hybridization signal (blue) in a ventral view onto the tube foot disc (**A**,**D**,**G**), in semi-thin sagittal section though the tube foot (B, E, H). The rectangle indicated in (**B**) is magnified in (**C**), the rectangle in (**E**) is magnified in (**F**). (I) shows the lateral part of the disc of a successive section of (**H**). s, stem; d, disc.

To further characterize the spatial expression patterns of adhesion candidate transcripts, we conducted semi-thin sections on selected in situ hybridized tube feet (Figure A5). Consistent with the whole mount sections, the semi-thin sections of TR61622_c8_g1_i2 showed strong labelling in the area where the adhesive secretory cell bodies were located (Figure A5A–C), whereas TR60905_c1_g1_i1 and TR55893_c4_g1_i1 presented a more disperse dotted expression pattern in the same disc area (Figure A5D–I).

## 3. Discussion

Temporary adhesion relies on the secretion of large proteins [29,30,39]. Amongst echinoderms, sea urchins and sea stars attach strongly but reversibly by means of specialized adhesive organs—the tube foot [36]. Not many commonalities have been found when it comes to their adhesive secretions [34,35]. A high conservation of the large cohesive protein sea star foot protein 1 (Sfp1) has recently been found for 18 sea star species, whereas the adhesive proteins were found to be less conserved [42]. Comparatively little is known for sea urchins. Adhesive (e.g., Nectin [45,46]) and releasing candidates, (e.g., endopeptidases [45]) have been proposed and they show some homologies with sea stars. However, with the increasing number of transcriptomic and proteomic data on non-permanent adhesives, some common features begin to emerge amongst taxonomically unrelated organisms that rely on reversible adhesion.

Mass spectrometry-based analyses have been frequently used in bioadhesion research [29,30,33,49,50,51,52] to identify the proteins involved in adhesive processes. The species investigated in the bioadhesion field are generally non-model organisms and lack the benefits of broad, commonly available datasets, such as transcriptomes and genomes. Analyzing proteome data without species-specific transcriptomic or genomic data is a difficult task, as mass spectrometry only provides short fragments of proteins and adhesive proteins often exceed a length of 500 amino acids [29,30,39]. The peptides derived from mass spectrometry are subsequently mapped against available databases, such as UniProt/Swiss-Prot with species-specific or less favorable, all protein entries of closely related species as settings. Unfortunately, a large quantity of database-derived proteins is in silico annotated, often lacking completeness and containing errors [53]. This leads to a discrepancy in which, depending on the quality and quantity of published proteins, a large set of peptides cannot be mapped or could be mapped to the wrong proteins, making the identification of adhesion-related proteins a daunting task. More favorable to protein identification via databases is the mapping of mass spectrometry-derived peptides to a species-specific transcriptome. This significantly increases the quantity of mapped peptides and strongly reduces the number of wrong protein assignments. New sequencing technologies, such as Oxford Nanopore or PacBio sequencing, can sequence long reads for a comparatively low price, which will improve transcriptome quality and completeness in future studies.

**Figure 7:**
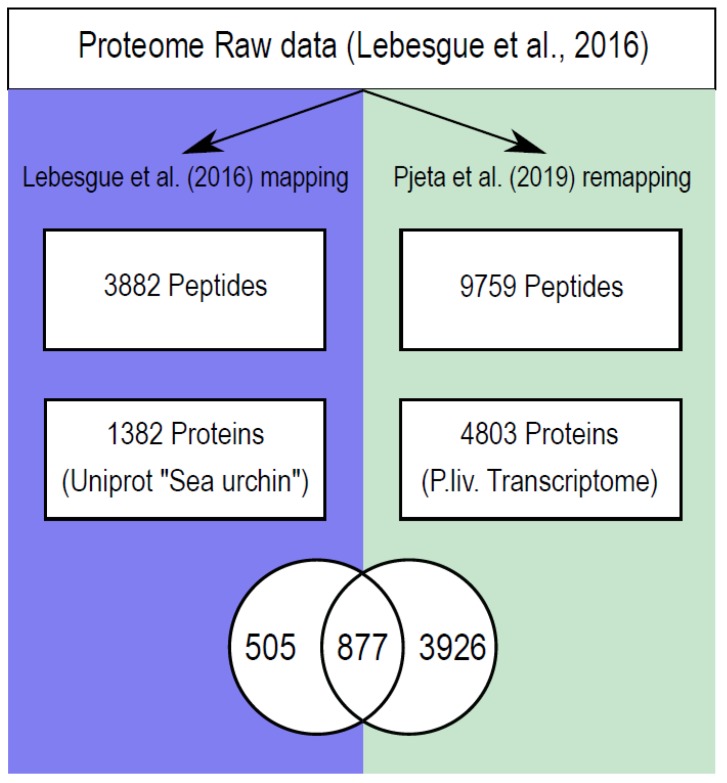
Comparison of the differential proteome re-analysis with the initial dataset of Lebesgue et al. Mass spectrometry peptide mapping to the transcriptome resulted in a drastic increase of successfully mapped peptides and proteins compared to the mapping of the previous study, in which the peptides were mapped against UniProt database hits for sea urchins. Both protein mappings had an overlap of 877 proteins, 505 are uniquely found in the Lebesgue data, whereas 3926 proteins are newly identified in the present study.

As a case study, we re-analyzed a proteomic dataset derived from the tube feet of *P. lividus*. In a previous study [45], potential adhesion candidates were identified through analyses of 21 samples, including tube foot adhesive discs and non-adhesive stems and samples of adhesive secretions. The mass spectrometry obtained peptides were mapped to sea urchin proteins of the UniProt database using the MASCOT algorithm [45]. In contrast to the previous study, we mapped all peptides against a *de novo*, species-specific tube feet transcriptome that resulted in a 60% increase in the mapped disc and stem peptides from 3882 in Lebesgue et al. [45] to 9759 mapped peptides in the present study (Figure 7). This resulted in an increase in identified proteins from 1382 UniProt-derived proteins to 4803 tube feet-specific transcripts. Interestingly, only 877 proteins were found in both mappings, whereas 505 proteins were exclusively found in Lebesgue et al. [45]. The latter can be explained by peptides being mapped to the wrong proteins because of amino acid variance between even closely related species. In this study, only tube feet RNA was used for transcriptome sequencing. Therefore, only genes that were expressed in the tube feet were represented in the transcriptome. Consequently, a subset of the proteins exclusively identified in Lebesgue et al. [45] could indeed be mapped to proteins not expressed in tube feet but in other parts of the sea urchin. As adhesive tissue is only present in tube feet discs, this issue should not interfere with the identification of adhesive proteins.

Following the successful proteome re-mapping, we combined these data with a differential RNAseq analysis of RNA reads derived from tube foot disc and stem tissue. As adhesive proteins are used for attaching the animals to a substrate during locomotion, adhesive proteins have to be constantly produced, leading to high gene expression levels. Therefore, by comparing the expression levels of genes from adhesive disc tissue with the non-adhesive stem tissues, we can possibly identify adhesion genes. This differential RNAseq approach has been performed for several species that are currently investigated in the bioadhesion community [29,30,33,54].

Combining the RNAseq data with the proteome narrows down the list of genes involved in adhesion as the proteome, especially the adhesive secretion dataset, delivers hard evidence that a protein is secreted. Furthermore, the adhesive disc tissue should be included in the investigation because it was shown that many secreted adhesives are often strongly cross-linked, making them insoluble for the proteases used prior to mass spectrometry [43,44,45]. In total, 121 proteins were identified to be overexpressed in the tube feet discs, they were also present in disc and/or adhesive secretion proteome datasets but not in the stem proteome.

Through a rigorous analysis of all candidate proteins, we were able to narrow down the number of candidate genes selected for in situ hybridization. Low-copy transcripts were excluded because it is highly unlikely that these genes have a major role in adhesion, as high amounts of adhesives must constantly be produced. Next, we excluded highly similar transcripts and putative isoforms because in situ hybridization probes also bind to messenger RNA with high similarity. Thus, in situ hybridization would give a chimeric expression pattern of two or more similar genes. In addition, all transcripts were checked for possible merging to larger sequences. It has been shown for the flatworm *M. lignano*, that large genes tend to be fragmented into several transcripts in short-read-based transcriptomes because of the large sequence size and the presence of repetitive regions [29].

Finally, conserved proteins, that are not likely to be involved in bioadhesion were identified by NCBI BLAST searches and excluded from the in situ screen. For instance, dynein is known to act as a motor- protein in intracellular vesicle transport [55]. In this study, dynein was identified several times in the differential RNAseq to be upregulated in tube feet discs. A possible explanation for the high dynein expression in discs is that there is also a high need for motor proteins for vesicle transport because of the high production and secretion of adhesives. Thus, dynein might play an indirect role in bioadhesion, but it is highly unlikely that it is a major adhesive component. Furthermore, proteins with a BLAST hit for spicule matrix proteins were also excluded. It has been shown that spicule matrix proteins are expressed in the primary mesenchymal cells of sea urchin embryos and have a role in skeletal formation [56,57]. Likewise, sea urchin tube feet contain a network-like calcareous skeleton [58]. Most likely, tube feet disc-specific spicule matrix proteins are involved in building up and maintaining skeletal structures, but they are not adhesion related and were therefore excluded from the in situ screen.

One more transcript, Nectin, was added to the ISH screen, although Nectin log2 differential expression in the disc did not meet our selection criteria. In the present differential gene expression analysis this protein was shown previously to be highly over-expressed in the disc and to be a part of the adhesive secretion [45,46].

In summary, through this stringent transcript evaluation process we reduced the number of transcripts from 121 to 49. Additionally, we selected 10 transcripts for in situ hybridization which had no hit in the proteome data. The selection process was highly similar to the proteome and differential transcriptome-based selection, but here, we aimed first for transcripts with high differential expression, combined with a large count of mapped reads in the disc. We also selected transcripts that contained conserved domains, which have been shown in previous studies to be parts of adhesive proteins (see Table 1). To our surprise, no adhesion-related expression pattern was identified in this transcriptome-only selection subset. All transcripts that showed adhesion-related expression patterns were differentially expressed in the discs and could be found in disc and adhesive secretion-only proteome datasets (Figure 6, Figure A1).

To identify which expression pattern could be considered adhesion related, we took advantage of adhesive proteins already characterized in sea stars. Recently, Lengerer et al. [42] published the sequences of 33 additional *A. rubens* adhesion candidate transcripts (Arub-1 to 33). We blasted these transcripts against our 59 adhesion candidates and found that 16 *A. rubens* sequences (including Sfp1) had significant similarity (*e*-value < 1 × 10^−5^) to eight *P. lividus* transcripts. In situ hybridization revealed a strong ring-shaped expression for six of these transcripts, indicating that this expression pattern is associated with adhesion-related tissue (two transcripts did not show a specific ISH pattern; Figure 6). A similar expression pattern was identified for 10 more transcripts of our in situ hybridization screen (Figure A1A–J). We conducted semi-thin sections of selected in situ hybridizations in order to further characterize the spatial distribution of the expression pattern. However, we were not able to distinguish between the different cell types because the tissue seemed to be damaged from the in situ hybridization process. A rough estimation of the spatial distribution of stained cells in the area of adhesive cell bodies could be made. Semi-thin sections also showed that during the hybridization process, non-adhesive epidermal tissue might possibly be lost. This could explain the relatively high number of in situ hybridizations with no expression pattern. In future in situ hybridization experiments, it is recommended that this be considered and that the duration of proteinase treatment be reduced.

The six transcripts that had an ISH expression pattern consistent with the location of the adhesive secretory cell bodies and simultaneously a sea star orthologue adhesion-related gene were identified to be Nectin, alpha-tectorin, uncharacterized protein, myeloperoxidase, neurogenic locus notch homologue protein and alpha-macroglobulin (Table S7).

TR60905_c1_g1_i1 was identified to be *P. lividus* Nectin (99% identity), but it shares some similarity with sea star Arub-27 (56.3% identity). Nectin has been identified to have been secreted into the adhesive material of *P. lividus* [45,46]. This protein contains six coagulation factor 5/8 C-terminal or discoidin domains that can bind galactose and *N*-acetylglucosamine residues and are usually found in extracellular and membrane proteins [45,46]. Four of these domains are also present in *A. rubens* Sfp1, which is believed to have a cohesive role because it is located at the fibrillar meshwork of the adhesive material and not on the homogenous priming film in contact with the substrate [39]. Thus, it can be hypothesized that Nectin is also a cohesive protein binding to free or conjugated galactose and *N*-acetylglucosamine residues within the adhesive material and/or that it is an adhesive protein, binding the adhesive material to the glycans present at the disc cuticle. Interestingly, two more transcripts (TR55893_c4_g1_i1 and TR51354_c0_g1_i3) presented the same dotted ring-shaped pattern but had no sea star orthologue adhesion-related gene. These transcripts were identified has sea urchin MSP130 protein that, similar to Nectin, is an embryonic cell surface glycoprotein [59] present in adult tube feet disc and adhesive secretion proteomic datasets. This seems to indicate that both proteins might have been co-opted in the adults to perform an adhesive or cohesive-related function.

Transcript TR63383_c2_g1_i1 was identified as sea urchin alpha-tectorin (76.2% identity) but shares partial sequence homologies with sea star Sfp1 (50% identity) and with Arub-4, -10 and -25 (26.7%, 51.3% and 51.4% identity). TR63383_c2_g1_i1 maps in the previous proteome [45] to a protein expressed only in the disc and the adhesive secretion, which agrees with sea star Sfp1, Arub-4 and -10 expression in the disc adhesive epidermis [42]. In *A. rubens,* Sfp1 is supposed to have a cohesive function [39]. It is a multi-conserved domain containing protein, which is translated from a single mRNA molecule and post-translationally cleaved into four subunits. TR63383_c2_g1_i1 presents in its sequence several domains that are recurrent in other marine adhesive and cohesive proteins. Domains such as von Willebrand factor type D domains, conserved cysteine residues, galactose-binding lectin domains, trypsin inhibitor-like cysteine rich domains and EGF-like domains are also present in the sequences of adhesive proteins not only from sea stars (Sfp1) but also from flatworms (Mlig-ap1 and -2, Mile-ap1 and Mile-ap2a/b) and cnidaria (Table 1), being associated with protein and carbohydrate-binding functions [29,30,33,40]. Proteins containing C- type lectin, EGF, vWF type A domains have also been implicated in the non-permanent adhesion of the defensive glue secreted by the terrestrial slug *Arion subfuscus* [60]. However, further investigations regarding the isolation and characterization of this protein in *P. lividus* need to be conducted.

TR43200_c3_g1_i5 has homology not only to a cephalochordate hypothetical protein (62.5% identity) but also to the sea star Arub-11 (32.4% identity). TR43200_c3_g1_i5 maps in the sea urchin previous proteome [45] to a protein expressed in the disc and the adhesive secretion, which is in agreement with the expression of Arub-11 in sea stars, which was only present in the disc adhesive epidermis [42]. The TR43200_c3_g1_i5 sequence contains trypsin inhibitor-like cysteine-rich domains, which typically contain 10 cysteine residues that form five disulphide bonds. This is in agreement with the reported insolubility of sea urchins and sea stars adhesive material, attributed to the presence of proteins with significant amounts of cysteines (2.6% and 3.2%, respectively) [34].

TR57217_c2_g1_i1 is similar to sea urchin myeloperoxidase (68.4% identity), as well as to sea star Arub-30 (39.9% identity). However, in sea urchin tube feet, TR57217_c2_g1_i1 was only expressed in the disc and the adhesive secretion, whereas in the sea star, Arub-30 is expressed in the tube feet disc and stem [42]. This is in line with the previous identification of peroxidase-like enzymes highly expressed in sea urchin tube foot discs, in sea star adhesive material and in cnidaria attachment basal area [33,38,45]. The present study thus provides further evidence of the role of peroxidases in some non-permanent adhesive systems, in which they might act as catalyzers of protein crosslinking within the adhesive, thus, contributing to its high cohesive strength.

TR63654_c4_g1_i1 was identified as sea urchin neurogenic locus notch homologue protein (77% identity), sharing also some resemblance with sea star Arub-1, -6, -20 and -24 (40.6%, 56%, 46% and 34.6%, respectively). TR63654_c4_g1_i1 and Arub-1, -6 and -20 are exclusively expressed in the disc [42]. The TR63654_c4_g1_i1 sequence contains domains, such as complement C1r/C1s, Uegf, Bmp1 (CUB) and calcium-binding EGF-like, which have calcium ion and protein binding abilities.

TR61622_c8_g1_i2 has homology with sea urchin alpha-2-macroglobulin-like protein (68.2% identity) and with sea star Arub-13 (35.8% identity). In sea urchins, TR61622_c8_g1_i2 maps to a protein expressed in the disc and the adhesive secretion, in accordance with the sea star Arub-13 that is specifically expressed in the adhesive disc epidermis [42]. Alpha-2-macroglobulin-like proteins are usually extracellular and alpha macroglobulin domains have been previously identified in barnacle cyprid larvae adhesive glycoproteins (Table 1) [18,19].

Amongst the 10 transcripts that had an ISH expression pattern consistent with the location of the adhesive secretory cell bodies, but no sea star orthologue adhesion-related gene, we identified three transcripts (TR52215_c0_g3_i6, TR58202_c1_g1_i1 and TR46467_c1_g1_i2) matching a mucin and two proteins with lectin-binding domains. Similar proteins were found in sea star adhesive secretions and considered adhesion candidates, given that mucin-like proteins have the ability to oligomerize and/or cross-link to other adhesive molecules forming structural networks, and lectin-like proteins can bind glycans within the adhesive present in the cuticle or in the substrate [38]. The remaining seven transcripts were identified as sea urchin MSP130 protein (TR55893_c4_g1_i1 and TR51354_c0_g1_i3) or uncharacterized proteins (TR50813_c1_g1_i4, TR50813_c1_g2_i1, TR46688_c0_g1_i1, TR35634_c1_g1_i1 and TR42843_c2_g1_i2) with any or little annotation (Table S7). Nevertheless, it should be noted that TR46688_c0_g1_i1 has the same domains present in TR63383_c2_g1_i1, which are recurrent in other marine adhesive proteins as discussed above.

In summary, our data show that it is highly recommended to integrate proteomic datasets with species-specific transcriptome data. Using this approach, we were able to improve the identification of proteins from a dataset previously published [45]. In a subsequent gene expression study, we were able to identify 16 genes that are possibly involved in the bioadhesion of *P. lividus*. These data can be used to generate novel biomimetic glues for industrial and medical purposes.

## 4. Materials and Methods

### 4.1. Specimen and Samples Collection

*Paracentrotus lividus* (Lamark 1816) were collected at low tide at Ericeira located in the western coast of Portugal (38.9756° N, 9.4203° W). They were brought to the laboratory and maintained in an aquarium with circulating artificial seawater (33 ppt, 16 °C) and fed with *Laminaria* sp., *Ulva* sp. and maize.

After 24 h of rearing in aquarium, sea urchins were placed upside down in containers filled with artificial seawater and their tube feet sectioned by the base of the stem close to the test and stored in Tri-Reagent (Sigma-Aldrich, St. Louis, MO, USA) at 4 °C. Some of these tube feet were previously dissected (working in Petri dishes placed on ice) to separate discs (tissue containing adhesive and de-adhesive cells) from stems (tissue without adhesive and de-adhesive cells) and then stored as above.

### 4.2. RNA Extraction

Total RNA was extracted from 60 tube feet (three replicates) collected from one individual for transcriptome sequencing and from 60 discs (three replicates) and 60 stems (three replicates) pooled from three individuals for differential gene expression analysis.

The samples were preserved in Tri-Reagent (Sigma-Aldrich T9424, St. Louis, MO, USA) and were mechanically homogenized using 1.4 mm ceramic beads in a Precellys Evolution homogenizer (Bertin Instruments, Montigny-le-Bretonneux, France). Homogenization settings were 2 × 30 s at 5000 rpm with a 20 s intermission.

RNA isolation was performed according to the manufacturer’s protocol followed by isopropanol precipitation. The RNA pellet was washed in 75% EtOH, centrifuged and subsequently air-dried. The pellet was dissolved in 20 µl nuclease –free water. Due to a high amount of co-precipitated tube foot pigments, we cleaned-up all RNA preparations using the One-Step PCR inhibitor removal Kit (Zymo Research, D6030, Irvine, CA, USA).

Additionally, tube foot RNA preparations that were used for *de-novo* transcriptome sequencing underwent a rigorous DNase treatment using the Turbo-DNA-free kit (Thermo Fisher Scientific, AM1907, Waltham, MA, USA) followed by a Sodium Acetate-Isopropanol precipitation. The amount and quality of RNA was assessed on a Nanodrop 2000c spectrophotometer (Thermo Fisher Scientific, Waltham, MA, USA).

### 4.3. Transcriptome and Differential Gene Expression

For transcriptome assembly, Illumina paired-end sequencing was performed on three libraries generated from three independent tube feet samples of one adult animal. From these libraries 29.515.426, 35.082.487 and 32.622.827 100 bp paired-end reads were obtained, respectively. Illumina reads were assembled using Trinity v2.0.4 [61] with default settings. Transcripts were clustered to 95% identity using cd-hit-EST software v.4.5.4 [62,63] to generate a low-redundant dataset for proteomics. The assembled transcriptome as well as the raw data files are available at the NCBI (Bioproject PRJNA602659).

For the identification of disc-specific transcripts, a differential gene expression analysis was performed. Three biological samples of tube foot disc tissue containing the adhesive and releasing glands and three biological samples of the tube foot stem tissue were collected. RNA was isolated using Tri Reagent. Illumina libraries were generated and 50 bp Illumina reads of the three disc samples (24.644.896, 17.793.830, 32.040.358) and the three stem samples (53.975.783, 32.793.628, 21.035.658) were sequenced. Reads were aligned against the transcriptome using bwa. Differentially expressed genes (false-discovery rate ≤ 0.05, with a minimum four-fold change) were identified using DESeq2 [64] and visualized using Instant Clue software suite [65].

### 4.4. Proteome Re-Mapping

Data analysis was performed using Proteome Discoverer 2.1 (Thermo Scientific) with search engine Sequest. The raw files were searched against the translated *P. lividus* transcriptome. Precursor and fragment mass tolerance was set to 10 ppm and 0.02 Da, respectively. Up to two missed cleavages were allowed. Carbamidomethylation of cysteine was set as static modification, and oxidation of methionine as variable modification of peptides. Acetylation, methionine-loss, and methionine-loss plus acetylation were set as N-terminal dynamic modification of proteins. Peptide identifications were filtered at 1% false discovery rate.

### 4.5. In situ Hybridisation

*In situ* hybridization probes were produced using gene-specific PCR products as a template. All primers used in this study are listed in Supplementary Material 4 Tab H and Supplementary Material 5 Tab C. Whole mount in situ hybridization was performed using a protocol initially developed for the flatworm *Macrostomum lignano* [66] with slight modifications. The tube feet of three different animals were collected and preserved in 4% paraformaldehyde in PBS overnight. After a de-and rehydration methanol series, tube feet were bleached in 5% formamide, 0.5× SSC, 1.2% H_2_O_2_ bleaching solution under bright light for 3 h [67]. Proteinase K digestion was performed at 37 °C for 15 min. After color development, the calcareous skeleton of the tube feet was dissolved using Morse’s solution, a formic acid-sodium citrate decalcification reagent [68]. After overnight incubation, tube feet were rinsed in PBS-0.001% Tween and mounted on a glass slide using Mowiol mounting medium. For a clearer view on the discs containing the adhesive tissue, the non-adhesive tube feet stems were cut off. Images of the tube feet discs were acquired using a Leica DM5000B Microscope.

### 4.6. ISH Semi-Thin Sections

Following a standard whole-mount in situ hybridization procedure, tube feet were preserved in BOUIN’s solution. After an increasing ethanol dehydration series, the samples were embedded in EMBed812. Two micrometer thick semi-thin sections were cut in series with a Reichert 2040 Autocut using a 6 mm Diatome Histobutler diamond knife. Series were mounted in cedar wood oil and examined with a Leica DM5000B microscope. Images were acquired with a DFC490 digital camera (Leica Microsystems, Wetzlar, Germany) and a Leica application suite 4.8. software.

### 4.7. Data Deposition

Sequencing raw data as well as the assembled transcriptome are available at NCBI, Bioproject PRJNA602659.

## Figures and Tables

**Table 1:** Common molecular features between non-permanent adhesives. Abbreviations: A2M—alpha macroglobulin domain; C8—cysteine-rich domain; C content—Cysteine content; CTL—C-type lectin domain; DS—Discoidin-like (F5/8 type C) domains; EGF—EGF-like calcium-binding domain; GRK rich-reg—Glycine, Arginine and Lysine rich region; T rich-reg.—Tyrosine rich region; TSP1—Thrombospondin 1 domain; TIL—trypsin inhibitor-like; VWD—von Willebrand domain.

Organism	Adhesive Organ	Protein Components						Glycan Components	Ref.
		Candidate Proteins			Characterised Adhesive Proteins				
		Adhesion	Polymerization	Release	Name (Accession) Function	*M*_W_s	Conserved Dom. Repeated Regions		
***Amphibalanus amphitrite***	Larvae-Antenullar discs	Basic and acidic prot. 20-kDa cement prot.			**SIPC** (AY423545) Adhesion, Settlement Biomineralization	171.7 kDa 3 subunits: 98, 88 and 76 kDa	A2M	Conjugated with a-linked mannose	[14,15,16,17,18]
***Balanus glandula***	Larvae-Antenullar discs				**MULTIFUNCin** (KC152471) Adhesion, Settlement, Biomineralization	199.6 kDa 3 subunits: 98, 88 and 76 kDa	A2M	Conjugated with a-linked mannose	[19]
***Macrostomum lignano***	Tail plate			Small negatively charged protein	**Mlig-ap1** (MH586844.1) Cohesion		CTL, VWD, TIL, C8, EGF. GRK rich-reg. 11% C content		[29]
					**Mlig-ap2** (MH586845.1) Adhesion		TIL, C8, vWD, TSP1	Conjugated with Gal-β(1–3)-GalNAc	
***Minona ileanae***	Tail plate				**Mile-ap1** (MK854810.1)			Probably O-glycosylated	[30]
					**Mile-ap2a/b** (MK854811.1 MK854812.1)		TSP1, TIL, T rich-reg.		
					**Mile-ap3a/b** (MK854813.1, MK854814.1)		GRK rich regions		
					**Mile-ap4** (MK854815.1)		P rich region		
					**Mile-ap5** (MK854816.1)		none		
***Hydra magnipapillata***	Basal disc	Transcript with chitin-binding domain	Peroxidase-like enzymes	Glycosyl hydrolase			vWD, C8, Gal, TIL, EGF		[33]
***Asterias rubens***	Oral tube foot disc	Proteins with hyalin, EGF, and discoidin domains	Peroxidase-like enzymes	Peptide hydrolase	**Sfp1** (X2KZ73) Cohesion	426 kDa 4 subunits: 57, 231, 72, 66 kDa	DS, vWD, CTL, C8, EGF, 5% C content	Free sialylated proteoglycans Conjugated Gal, GalNAc, fucose, sialic acid residues	[37,38,39,40]
***Asterina gibbosa***	Oral tube foot disc							α-linked mannose residues	[41]
***Paracentrotus lividus***	Oral tube foot disc		Peroxidase-like enzymes	Peptide and glycosyl hydrolases	**Nectin-variant 2** (A0A182BBB6) Adhesion	108.3 kDa	DS; 1.1% C content		[43,44,45,46]

**Table 2:** Summary of the *P. lividus* transcriptome assembly.

Total number of transcripts	270,361
Number of transcripts after CD-HIT 95% clustering	182,027
Total length (bp)	225,573,582
Longest transcript (bp)	11,173
Shortest transcript (bp)	224
Average transcript length (bp)	834.34
N50 length (bp)	1499
Percentage GC	37.26

## Supplementary Materials

Supplementary Materials can be found at https://www.mdpi.com/1422-0067/21/3/946/s1.

## Abbreviations

ISH	*In situ* hybridization
BLAST	Basic Local Alignment Search Tool
NCBI	National Center for Biotechnology Information
